# Cognitive Function in Childhood and Lifetime Cognitive Change in Relation to Mental Wellbeing in Four Cohorts of Older People

**DOI:** 10.1371/journal.pone.0044860

**Published:** 2012-09-10

**Authors:** Catharine R. Gale, Rachel Cooper, Leone Craig, Jane Elliott, Diana Kuh, Marcus Richards, John M. Starr, Lawrence J. Whalley, Ian J. Deary

**Affiliations:** 1 MRC Lifecourse Epidemiology Unit, University of Southampton, Southampton, United Kingdom; 2 Centre for Cognitive Ageing and Cognitive Epidemiology, Department of Psychology, University of Edinburgh, Edinburgh, United Kingdom; 3 MRC Unit for Lifelong Health and Ageing, University College London, London, United Kingdom; 4 Institute of Applied Health Sciences, University of Aberdeen, Aberdeen, United Kingdom; 5 Centre for Longitudinal Studies, Institute of Education, University of London, London, United Kingdom; 6 Geriatric Medicine Unit, University of Edinburgh, Royal Victoria Hospital, Edinburgh, United Kingdom; University of Granada, Spain

## Abstract

**Background:**

Poorer cognitive ability in youth is a risk factor for later mental health problems but it is largely unknown whether cognitive ability, in youth or in later life, is predictive of mental wellbeing. The purpose of this study was to investigate whether cognitive ability at age 11 years, cognitive ability in later life, or lifetime cognitive change are associated with mental wellbeing in older people.

**Methods:**

We used data on 8191 men and women aged 50 to 87 years from four cohorts in the HALCyon collaborative research programme into healthy ageing: the Aberdeen Birth Cohort 1936, the Lothian Birth Cohort 1921, the National Child Development Survey, and the MRC National Survey for Health and Development. We used linear regression to examine associations between cognitive ability at age 11, cognitive ability in later life, and lifetime change in cognitive ability and mean score on the Warwick Edinburgh Mental Wellbeing Scale and meta-analysis to obtain an overall estimate of the effect of each.

**Results:**

People whose cognitive ability at age 11 was a standard deviation above the mean scored 0.53 points higher on the mental wellbeing scale (95% confidence interval 0.36, 0.71). The equivalent value for cognitive ability in later life was 0.89 points (0.72, 1.07). A standard deviation improvement in cognitive ability in later life relative to childhood ability was associated with 0.66 points (0.39, 0.93) advantage in wellbeing score. These effect sizes equate to around 0.1 of a standard deviation in mental wellbeing score. Adjustment for potential confounding and mediating variables, primarily the personality trait neuroticism, substantially attenuated these associations.

**Conclusion:**

Associations between cognitive ability in childhood or lifetime cognitive change and mental wellbeing in older people are slight and may be confounded by personality trait differences.

## Introduction

Poorer cognitive function in youth is a risk factor for common mental health problems many years later. Children or adolescents who score higher on tests of intelligence are less likely to be diagnosed with depressive or anxiety disorders or to report symptoms of psychological distress later in life. [1]–[6] Such problems represent only one extreme of the broad spectrum of mental health. According to Keyes, in the majority of the general population who are not mentally ill there is wide variation in levels of mental health, with some people ‘flourishing’ (enthusiastic about life and actively engaged with other people), others ‘languishing’ (‘a life of quiet despair’) [7], and the remainder ‘moderately mentally healthy’. [8] Keyes' view that mental health should be regarded not just as the absence of mental illness but as a state of complete emotional, psychological and social wellbeing is part of a growing international interest in what has come to be called positive mental health, often referred to as mental wellbeing. [8], [9] Many researchers now agree that mental wellbeing is best thought of as a multi-faceted phenomenon, involving not just positive feelings such as happiness or contentment, but also positive functioning whereby individuals behave in ways that provide engagement and fulfilment. Whether cognitive function in youth is predictive of mental wellbeing later in life is largely unknown.

Cognitive function at older ages is substantially determined by peak cognitive ability attained in young adulthood and also reflects the change in ability since that time. [10] According to Rowe & Kahn's model of successful ageing, the maintenance of high levels of cognitive function is a crucial part of ageing well. [11] Older people with better cognition are likely to be more engaged with life [12] which itself is a determinant of feelings of happiness and contentment though the direction of causation between engagement and cognition in old age is debatable. [13] Moreover, some evidence suggests that individuals' cognitive abilities in adulthood may bear little or no relation to how happy or satisfied they are with their lives. Most such studies, largely cross-sectional in design, have been based on small, unrepresentative samples and have not specifically studied older people. [14] In two longitudinal studies of people aged 70 or over neither cognitive ability in youth, [15] increases in cognitive limitations in later life, [16] nor extent of cognitive change since childhood [15], [16] were significantly associated with feelings of happiness or life satisfaction.

**Table 1:** Characteristics^1^ of the participants from the ABC1936, LBC1921, NCDS and NSHD.

	ABC1936 n = 142	LBC1921 n = 172	NCDS n = 6546	NSHD n = 1331
WEMWBS score	54.6 (7.28)	49.9 (8.01)	49.6 (7.85)	51.7 (7.96)
Age at wellbeing assessment	73.8 (0.81)	86.6 (0.43)	50.7 (0.15)	63.6 (0.76)
Cognitive ability at age 11				
MH test score	46.2 (11.0)	48.1 (11.2)	-	-
NFER test score	-	-	46.8 (14.8)	48.6 (14.7)
Father in professional or managerial social class	30 (21.1)	72 (41.9)	1722 (26.3)	330 (24.8)
Educational attainment				
Diploma/Degree	-	-	1371 (21.1)	323 (24.2)
Years in full-time education,	11.5 (2.27)	11.2 (2.51)	-	-
Professional or managerial social class	76 (53.5)	122 (70.9)	3107 (47.5)	632 (47.5)
Neuroticism				
IPIP	-	25.8 (7.65)	28.7 (7.11)	-
NEO	16.1 (7.10)	-	-	-
Pintner	-	-	-	9.50 (4.31)
Extraversion				
IPIP	-	20.7 (7.41)	29.4 (6.63)	-
NEO	28.0 (5.78)	-	-	-
Pintner	-	-	-	8.66 (2.35)
Cognitive ability in later life				
Ravens matrices	39.5 (7.06)	33.6 (7.55)	-	-
Logical memory	-	35.2 (13.4)	-	-
Verbal fluency^2^	-	42.8 9 (11.4)	22.7 (6.22)	24.4 (6.98)
Word recall^3^	-	-	6.63 (1.44)	24.7 (6.02)
Letter search speed^2^	-	-	334.9 (87.1))	345 (75.2)
Age at cognitive testing	64.5 (0.73)	79.1 (0.58)	50.7 (0.15)	53.4 (0.17)
Chronic disease in later life				
Cardiovascular disease^4^	14 (9.85)	30 (17.4)	-	62 (4.66)
Diabetes	3 (2.11)	2 (1.2)	239 (3.65)	28 (2.10)
Hypertension	34 (23.9)	72 (41.9)	923 (14.1)	172 (12.9)
Health limits activities	-	-	755 (11.5)	-

1 Values are means (SD) or number (percentage). ^2^Verbal fluency was assessed using a single timed test of animal naming in NCDS and NSHD and using three timed tests of generation of words beginning with C, F and L in LBC192121, hence the higher scores in the latter cohort. ^3^The two cohorts that used the word recall test employed different protocols and scoring, hence the variation in mean scores. ^4^For ABC1936, information on cardiovascular disease at the time of cognitive testing was restricted to heart disease.

Recent years have seen the development of a new measure of mental wellbeing, The Warwick-Edinburgh Mental Wellbeing Scale, that focuses entirely on positive feelings and positive functioning. [17] Members of four birth cohorts from the United Kingdom recently completed this scale at ages ranging from 50 to 87 years. These cohorts are exceptional in that their members took tests of cognitive ability in childhood and again in later life, making it possible to assess how their cognitive abilities have changed over the intervening decades. We took the rare opportunity these data offer to investigate whether cognitive ability at age 11 years, cognitive ability in later life, or lifetime cognitive change were associated with mental wellbeing in older people.

## Methods

HALCyon – Healthy Ageing across the Life Course – is a collaborative research programme using data from nine UK cohorts to examine how factors across the life course influence mental wellbeing and other aspects of healthy ageing in older people. This study uses data from the four HALCyon cohorts that have information on cognition both in childhood and in later life.

**Figure 1:**
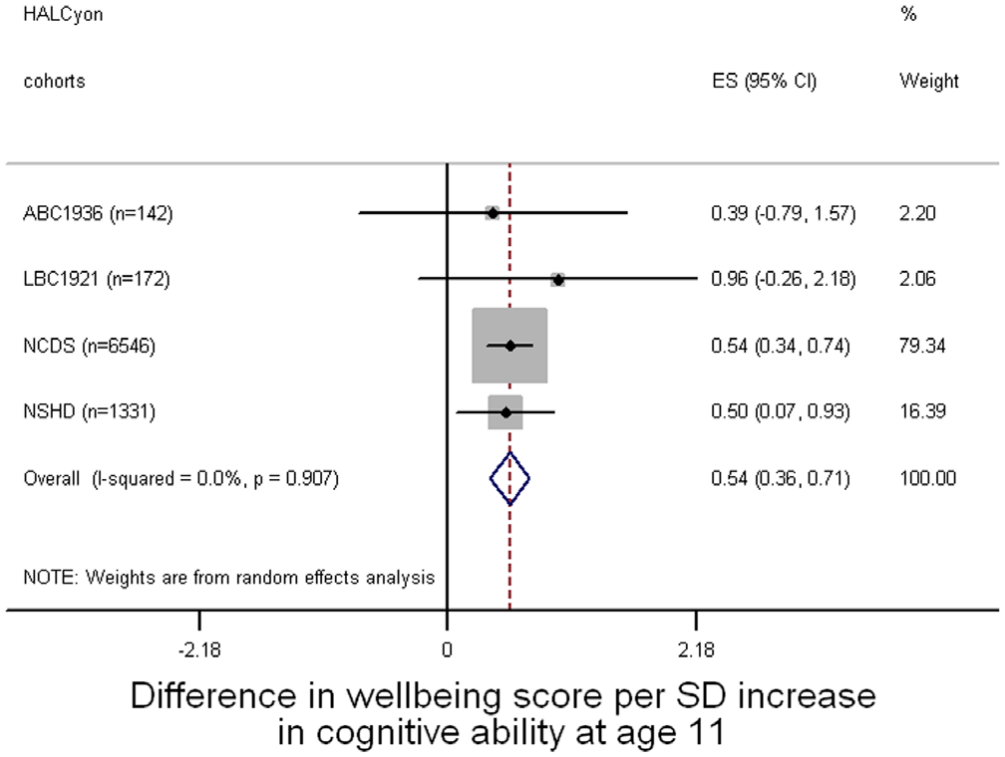
Forest plot of meta-analysis of the relationship between cognitive ability at age 11 and mental wellbeing in later life. ES = effect size (difference in mental wellbeing score).

**Figure 2:**
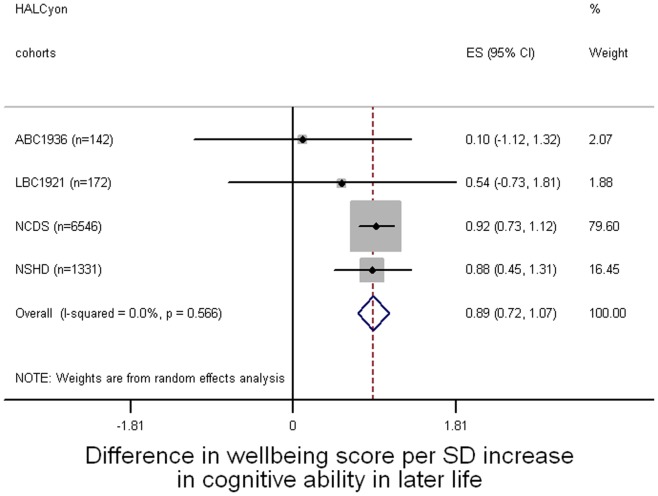
Forest plot of meta-analysis of the relationship between cognitive ability in later life and subsequent mental wellbeing.

**Figure 3:**
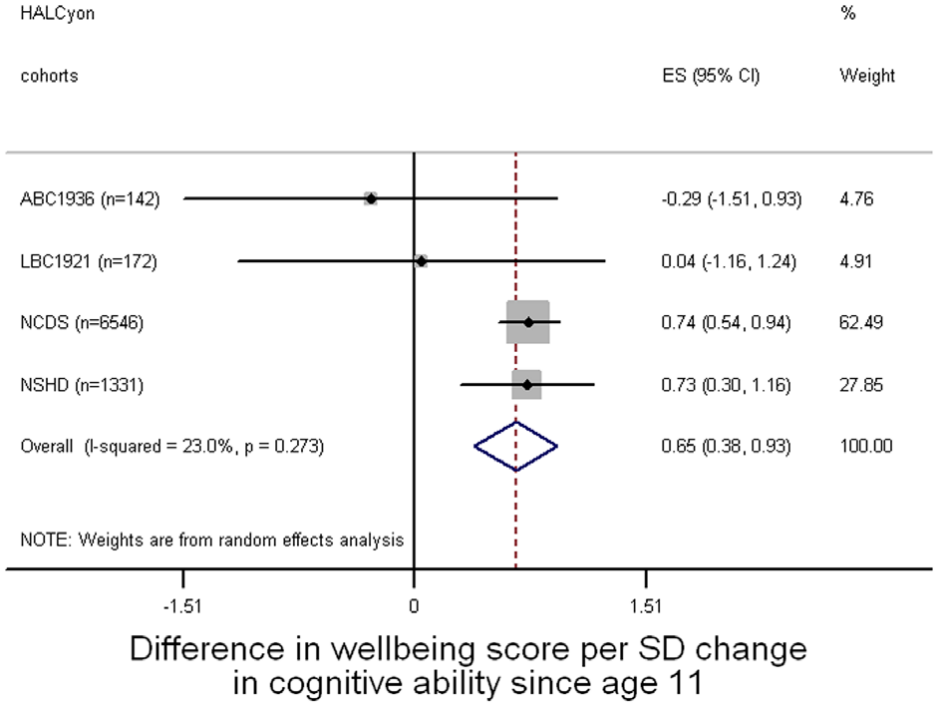
Forest plot of meta-analysis of the relationship between lifetime cognitive change and subsequent mental wellbeing.

### Ethics statement

Written informed consent was obtained from the parents for childhood measurements and ethical approval for the adult data collection was obtained from the Multicentre Research Ethics Committee for Scotland, the South East Multicentre Research Ethics Committee, and the North Thames Multicentre Research Ethics Committee.

### The Aberdeen Birth Cohort 1936 (ABC1936)

In 1947, as part of the Scottish Mental Survey, 70,805 children born in 1936 who attended school in Scotland sat a test of mental ability, the Moray House Test number 12. In 1999–2001, 567 of these people living in the Aberdeen area, then with a mean age of 64.4 years, were invited to participate in a study on cognitive ageing. [18] Participants were followed up subsequently and completed the Warwick-Edinburgh Mental Wellbeing Scale (WEMWBS) at a mean age of 73.9 years.

### The Lothian Birth Cohort 1921 (LBC1921)

In 1932, the Scottish Mental Survey tested the mental ability of 87,498 children born in 1921 who attended school in Scotland using the Moray House Test number 12. In 1999–2001, surviving members of this survey living in the Edinburgh area were invited to participate in a study on cognitive ageing. [18] In total, 550 people, then aged 79 years, took part in this LBC1921 study. Participants were followed up subsequently and completed the WEMWBS at a mean age of 86.6 years. [10].

**Table 2:** Results of meta-analyses estimating the overall difference in mental wellbeing score points for a standard deviation increase in cognitive ability at age 11, cognitive ability in later life and lifetime cognitive change.

	Adjustments^1^	Overall effect	Heterogeneity^2^
Cognitive ability at age 11			
	Unadjusted	0.53 (0.36, 0.71)	*I^2^* = 0% (0%, 17%), p = 0.91
	Socioeconomic position in childhood	0.49 (0.31, 0.67)	*I^2^* = 0% (0%, 28%), p = 0.89
	Educational attainment	0.21 (0.02, 0.40)	*I^2^* = 0% (0%, 81%), p = 0.49
	Socioeconomic position in later life	0.28 (0.10, 0.46)	*I^2^* = 0% (0%, 52%), p = 0.81
	Neuroticism	0.19 (0.04, 0.33)	*I^2^* = 0% (0%, 73%), p = 0.64
	Extraversion	0.43 (0.27, 0.59)	*I^2^* = 0% (0%, 63%), p = 0.74
	Chronic disease	0.47 (0.31, 0.63)	*I^2^* = 0% (0%, 0%), p = 0.93
Cognitive ability in later life			
	Unadjusted	0.89 (0.72, 1.07)	*I^2^* = 0% (0%, 77%), p = 0.57
	Socioeconomic position in childhood	0.87 (0.69, 1.04)	*I^2^* = 0% (0%, 76%), p = 0.59
	Educational attainment	0.69 (0.42, 0.93)	*I^2^* = 14% (0%, 87%), p = 0.32
	Socioeconomic position in later life	0.71 (0.54, 0.89)	*I^2^* = 0% (0%, 76%), p = 0.59
	Neuroticism	0.47 (0.06, 0.88)	*I^2^* = 55% (0%, 85%), p = 0.08
	Extraversion	0.65 (0.42, 0.87)	*I^2^* = 15% (0%, 87%), p = 0.32
	Chronic disease	0.83 (0.65, 1.02)	*I^2^* = 2% (0%, 85%), p = 0.38
Lifetime cognitive change			
	Unadjusted	0.66 (0.39, 0.93)	*I^2^* = 23% (0%, 88%), p = 0.27
	Socioeconomic position in childhood	0.68 (0.50, 0.87)	*I^2^* = 1% (0%, 84%), p = 0.39
	Educational attainment	0.53 (0.21, 0.86)	*I^2^* = 36% (0%, 77%), p = 0.20
	Socioeconomic position in later life	0.59 (0.38, 0.81)	*I^2^* = 7% (0%, 86%), p = 0.35
	Neuroticism	0.27 (−0.21, 0.77)	*I^2^* = 69% (13%, 89%), p = 0.02
	Extraversion	0.46 (0.18, 0.74)	*I^2^* = 28% (0%, 73%), p = 0.24
	Chronic disease	0.63 (0.37, 0.90)	*I^2^* = 21% (0%, 88%), p = 0.28

1 Adjustments were made for each covariate separately. ^2^ *I^2^* statistic with 95% confidence intervals, p-values from Cochran's *Q* statistic.

### The National Child Development Survey (NCDS)

The National Child Development Study (1958 cohort) was originally based on over 17,000 live births in Great Britain during one week in 1958. [19] The cohort has been followed-up through childhood and adult life. Participants completed the WEMWBS at a mean age 50.7 years.

### The MRC National Survey of Health and Development (NSHD)

The MRC National Survey of Health and Development (1946 cohort) grew out of a maternity survey of all mothers who had a baby in England, Scotland, or Wales in one week in March 1946. The cohort was originally based on 5,362 participants and has been followed-up through childhood and adult life. [20] Participants completed the WEMWBS at a mean age of 63.6 years.

### Cognitive ability in childhood

Cognitive ability of all cohort members was assessed at school at age 11 years. Members of the ABC1936 and LBC1921 cohorts took a general cognitive ability test, a version of the Moray House Test number 12. This test was validated against the Stanford Revision of the Binet Scale (r = 0.80) [21].

Children in the NCDS and NSHD took a general ability test, devised by the National Foundation for Educational Research in England and Wales. [22] Scores correlate strongly with scores on a test of verbal ability used to select 11-year-old children for secondary school (r = 0.93) suggesting high validity. [22].

### Cognitive ability in later life

In the ABC1936 fluid intelligence was assessed at a mean age of 64 years using a test of non-verbal reasoning, Raven's Standard Progressive Matrices. [23] Members of the LBC1921 took three tests of cognitive ability at a mean age of 79 years: Raven's Standard Progressive Matrices; [23] verbal fluency (producing as many words as possible beginning with C, F, and L); [24] and logical memory (immediate and delayed recall of two short stories), part of the Wechsler Memory Scale-IIIUK. [25] Cognitive impairment among members of the ABC1936 and LBC1921 was assessed using the Mini Mental State Examination. NCDS cohort members took four tests of cognitive ability at a mean age of 50.7 years: verbal fluency (animal naming), memory (word list recall and delayed word list recall – based on 10 items), and visual processing speed (letter cancellation). Three similar tests were taken by the NSHD cohort members at age 53 years: verbal fluency (animal naming), memory (a 3-trial 15-item word list), and speed and concentration (timed letter search).

### Mental wellbeing in later life

Mental wellbeing was assessed using the Warwick-Edinburgh Mental Wellbeing Scale (WEMWBS). [17] This scale was developed to measure a wide conception of mental wellbeing, including positive affect, psychological functioning (autonomy, competence, self acceptance, personal growth) and interpersonal relationships, and to be suitable for monitoring mental wellbeing at a population level. Confirmatory factor analysis suggests it measures a single underlying concept. [17] It has been validated on a representative general population sample of adults. The scale consists of 14 positively-worded statements. Examples include ‘I’ve been feeling optimistic about the future', ‘I’ve been feeling interested in other people', ‘I’ve been dealing with problems well', ‘I’ve been feeling good about myself', ‘I’ve been feeling useful'. For each statement, respondents are asked to indicate which of five options, ranging from none of the time (score 1) to all of the time (score 5), best describes their experience over the last two weeks. The overall score is calculated by summing the scores for each item. A higher score indicates a higher level of mental wellbeing. A few participants (<1%) in NSHD, NCDS and LBC1921 had missing data on one or more items. For those individuals with up to three missing items, we imputed an overall score by taking the score attained on the completed items and adding to this their mean score multiplied by the number of missing items. Individuals with more than three missing items were excluded. The Cronbach alpha for the 14 items in the four cohorts was 0.89 (ABC1936 and LBC1921) or 0.91 (NSHD and NCDS) showing high internal consistency.

### Covariates

We selected as potential confounding or mediating variables those factors that have been associated in previous studies with cognitive ability in childhood and later life and scores on the WEMWBS or other measures of subjective wellbeing and for which data were available in all four cohorts. These factors were: socioeconomic position in childhood and later life, [26]–[30] educational attainment, [27], [31]–[33] the personality traits neuroticism and extraversion, [27], [32], [34]–[36] and physical health. [37]–[40].

Socioeconomic position in childhood was defined using father's occupational social class. In ABC1936 and LBC1921 participants provided information on their father's occupation when they were age 11 years. In NCDS and NSHD, information was collected on father's occupation during the 11-year follow-up interviews with parents. Socioeconomic position in later life was defined using occupational social class reported at age 50 (NCDS), age 53 (NSHD), age 64 (ABC1936) and age 79 (LBC1921). In LBC1921 this was based on their own highest ranked occupation (or for married women, their husband's). In all other cohorts this was based on their own (or for married women in ABC1936, their husband's) current or most recent occupation. Occupations were categorized according to the Registrar General's classification (LBC1921, NCDS and NSHD) or the Standard Occupational Classification (ABC1936). For those members of NCDS or NSHD who had missing social class data at age 50 or 53 respectively we used data on social class from preceding follow-ups. Educational attainment was defined as highest academic qualification and their vocational equivalent (ABC1936, NCDS and NSHD) or number of years in full-time education (LBC1921). In three of the cohorts, the personality traits neuroticism and extraversion were assessed at the same time as later life cognitive ability using the relevant items from NEO Five Factor Inventory [41] (ABC1936) or the IPIP Big-Five Factor Inventory (LBC1921 and NCDS). [42] In NSHD, neuroticism and extraversion were assessed at the age of 13 years using Pintner's Aspects of Personality Inventory. [43] The cohorts differed in the data available on physical health at the time cognitive ability was measured in later life. For this analysis, we used data on reported history of diabetes, hypertension and cardiovascular disease (or heart disease only in the case of ABC1936). No data on history of hypertension was available for NSHD at age 53 so we used use of anti-hypertensive drugs as a proxy. No data on history of cardiovascular disease were available for the 50-year-old NCDS members; for this cohort, we used data on whether health limited their everyday activities.

### Analytical samples

In total, 11,131 men and women completed the WEMWBS (189 from ABC1936, 232 from LBC1921, 8745 from NCDS and 1965 from NSHD). Our analyses are based on 8191 people who had data on cognitive ability at age 11 and in later life and all the covariates. We excluded from our analyses a few individuals (n = 13) in the two oldest cohorts (ABC1936 and LBC1921) who had Mini-Mental State Examination scores less than 24. The analytical sample represents 74% of those who completed the scale (68% in NSHD, 74% in LBC1921 and 75% in NCDS and ABC1936). There were no differences in mental wellbeing score between the people in our analytical sample and those who were excluded due to missing data. Cognitive ability at age 11 and in later life tended to be poorer in the people who completed the WEMWBS but were excluded from our sample due to missing data, but these differences were in general small (≤0.1 of a standard deviation in the case of cognitive ability in later life and ≤0.2 of a standard deviation in the case of cognitive ability at age 11).

### Statistical analysis

We converted scores on the tests of general cognitive ability taken by all the cohorts in childhood and on the fluid intelligence test (Raven's Progressive Matrices) taken by the ABC1936 in later life into standard scores (mean  = 0; SD  = 1). For the other three cohorts a standardized overall measure of general cognitive ability in later life was generated by applying principal components analysis (PCA) to the test scores from each cohort separately and extracting (and calculating a score for each person on) the first unrotated principal component that reflects the variance shared among the tests taken. A general cognitive ability factor typically accounts for around 50% of the variance when a diverse battery of cognitive tests are given to a healthy population sample. [44] In LBC1921 this component accounted for 52.3% of the variance; loadings of the three tests on the factor were 0.62 (verbal fluency), 0.73 (logical memory) and 0.80 (Raven's matrices). In NCDS, this component accounted for 46% of the variance; loadings of the tests on the factor were 0.61 (verbal fluency), 0.84 (word list recall immediate), 0.82 (word list recall delayed), 0.25 (letter search speed). In NSHD, this component accounted for 50% of the variance; loadings of the tests on the factor were 0.76 (verbal fluency), 0.78 (word list recall), 0.56 (letter search speed).

To calculate the degree of lifetime cognitive change, we carried out a linear regression analysis in each cohort in which cognitive ability at age 11 was the independent variable and cognitive ability in later life was the dependent variable, and saved the standardized residual as a measure of cognitive change.

We used linear regression to examine the association between cognitive ability in childhood, cognitive ability in later life, and lifetime cognitive change (all expressed as SD scores) and scores on the WEMWBS. To obtain an overall estimate of the effect of each cognitive ability measure and to quantify the uncertainty of that estimate, we used meta-analysis to combine the estimates from each cohort. We calculated the pooled effect of each potential risk factor using DerSimonian and Laird random effect models, thereby incorporating an estimate of between-sample variation into the calculation. [45] We examined the heterogeneity of the estimates between the samples using *I* ^2^ (with 95% confidence intervals) and *Q* statistics. [46] The *I^2^* statistic provides the percentage of total variation across studies due to heterogeneity rather than chance. Figures for *I^2^* of 25%, 50% or 75% suggest low, moderate or high heterogeneity respectively. [46] We produced forest plots to describe the results of each meta-analysis. Finally, we examined how our findings changed if meta-analyses were based on effect estimates that had been separately adjusted for the covariates (socioeconomic position in childhood or later life, educational attainment, the personality traits, neuroticism and extraversion and presence of chronic disease).

## Results

Preliminary analyses showed that there were no significant differences in mental wellbeing scores between men or women in any of the cohorts – a finding consistent with other studies [14] – and the relation between each of our measures of lifetime cognition and mental wellbeing did not differ significantly between the sexes in any of the cohorts. We therefore analysed men and women together.

Table 1 shows the characteristics of the participants. Scores on the Warwick Edinburgh Mental Wellbeing Scale were normally distributed in each of the cohorts. Scores spanned the whole potential range (14 to 70) in the youngest cohort, NCDS, and were narrower in older cohorts: 18 to 70 in NSHD, 37 to 70 in ABC1936, and 24 to 68 in LBC1921. There was no clear pattern between mental wellbeing and the age of the cohorts. Within the narrow age range in each cohort there was no difference in mental wellbeing scores by age. The correlation between cognitive ability at age 11 and cognitive ability in later life was 0.42 in ABC1936, 0.43 in NCDS, 0.51 in LBC1921, and 0.53 in NHSD.

Figures 1–3 present forest plots of the meta-analyses of unadjusted estimates of the overall difference in mental wellbeing score for a standard deviation increase in cognitive ability at age 11, cognitive ability in later life, and lifetime cognitive change respectively. Better cognitive performance at age 11 (Figure 1) or in later life (Figure 2) was associated with higher mental wellbeing scores. Cognitive change between age 11 and later life was also associated with mental wellbeing: people whose later life cognitive performance was relatively better than their cognitive ability at age 11 tended to have higher mental wellbeing (Figure 3). All these effect sizes were small: a one standard deviation increase in cognitive ability in youth, in later life, or in cognitive change between these time points was associated with an increase in mental wellbeing of about 0.1 of a standard deviation.

We repeated our regression analyses adjusting separately for potential confounding or mediating factors. Table 2 shows the overall estimates of effect from all the meta-analyses, both unadjusted and adjusted separately for each covariate. The association between cognitive ability in childhood and mental wellbeing was substantially attenuated by adjustment for neuroticism: before adjustment the advantage in mental wellbeing score for a standard deviation higher score in childhood cognitive ability was 0.53 (0.36, 0.71); the equivalent figure after adjustment was 0.19 (0.04, 0.33). Adjustment for educational attainment or socioeconomic position in later life also weakened the association markedly, but adjustment for extraversion, socioeconomic position in childhood or chronic disease in later life had much smaller attenuating effects. Neuroticism also had a strong attenuating effect on the association between cognitive ability in later life and mental wellbeing: before adjustment the advantage in mental wellbeing score for a SD increase in cognitive ability was 0.89 (0.72, 1.07), after adjustment it was 0.47 (0.06, 0.88). Adjustment for extraversion, educational attainment or socioeconomic position in later life had small attenuating effects, reducing the effect size by less than a third. As before, the association was only slightly weakened by adjustment for socioeconomic position in childhood or chronic disease in later life. The association between lifetime cognitive change and mental wellbeing was also attenuated substantially by adjustment for neuroticism: the effect size was reduced by over half, changing from 0.66 (0.39, 0.93) to 0.27 (−0.21, 0.77). Adjustment for extraversion reduced the effect size by over a third. Educational attainment and later life socioeconomic position had smaller attenuating effects. The association changed very little or was even slightly strengthened, when adjusted for chronic disease in later life or childhood socioeconomic position respectively.

For most of the overall effect estimates the *I^2^* statistics were between 0% and 36% implying low or moderate heterogeneity. The exceptions were the effect estimates adjusted for neuroticism where values for *I^2^* were markedly higher. However, as expected given the small number of our samples, 95% confidence intervals around the *I^2^* statistics tended to be wide, ranging from 0% to as high as 89%, suggesting considerable uncertainty as to the true extent of heterogeneity.

## Discussion

In meta-analyses of data from over 8000 older men and women higher cognitive ability in childhood or in later life was associated with greater mental wellbeing. There was also an association between lifetime cognitive change and mental wellbeing: levels of wellbeing were slightly higher in people whose later life cognitive ability was greater than would be expected given their cognitive performance in childhood. These effect sizes were small and were substantially attenuated after adjustment for the personality trait neuroticism. Adjustment for the personality trait extraversion, educational attainment or socioeconomic position in later life also had attenuating effects on the associations, but they were little changed by adjustment for childhood socioeconomic position and chronic disease in later life.

Previous studies have shown fairly consistently that poorer cognitive ability in youth is linked with an increased risk of mental illness or symptoms of anxiety and depression later in life. [1]–[6] Our findings here suggest that cognitive ability in childhood may be less strongly predictive of mental wellbeing. Overall estimates from meta-analyses showed that the advantage in the mean Warwick Edinburgh Mental Wellbeing score for a standard deviation higher score in childhood intelligence was about half a point which represents approximately 0.1 of a standard deviation. That association seemed to be partly mediated through socioeconomic position in later life or educational attainment, themselves in part attributable to childhood cognitive ability, [31], [47] but also substantially and more interestingly confounded by the personality trait neuroticism. Extraversion too played some part in the association, but its role was markedly smaller than that of neuroticism. There were slightly larger associations between cognitive ability in later life and lifetime cognitive change and mental wellbeing that also appeared to be substantially confounded by neuroticism. These findings raise the question of whether the associations found in previous studies between cognitive ability in youth and risk of mental illness might also be due in part to confounding by personality. Neuroticism consistently shows a small inverse correlation with cognitive ability. [27], [34], [36], [48] Findings on the relationship between extraversion and cognition have been less consistent, [48] but two studies have found small positive correlations, [34], [36] Both neuroticism and extraversion are powerful influences on wellbeing. [35], [49] One large study of middle-aged twins estimated that socioeconomic position, educational attainment, income and marital status each explained less than 3% of the variance in wellbeing, while up to half the variance in how happy people felt was due to heritable personality traits. [50].

To our knowledge, only two published studies have investigated whether change in cognition is linked with how happy or satisfied older people are with their lives. One study of people aged 70 and over found no evidence that increases in cognitive limitations, as measured by the Mini Mental State Examination, over a 54 month period influenced the trajectory of positive affect or happiness; [16] this test is crude by comparison with the lifetime measure of cognitive change that we used in the present investigation. The other study, of the LBC1921 cohort at age 79, found no association between change in cognitive ability since childhood and life satisfaction. [15] The Warwick Edinburgh Mental Wellbeing scale differs from measures that assess happiness or life satisfaction alone in that it incorporates items on psychological functioning (autonomy, competence, self acceptance, personal growth) and interpersonal relationships. In our meta-analysis we found that people whose later life cognitive function was better than expected given their abilities in childhood did have significantly higher scores on this scale. This difference was modest and it was substantially attenuated such that it ceased to be statistically significant after adjustment for neuroticism and extraversion.

The observation in these cohorts that childhood socioeconomic position appeared to play little part in the associations between our measures of lifetime cognitive ability and mental wellbeing is consistent with previous findings that the association between greater cognitive ability in childhood and reduced risk of mental disorders or symptoms of psychological distress later in life is not confounded by parental social background. [2], [3], [5], [6].

Our finding that adjusting for chronic disease had only a small attenuating effect on the associations between cognitive ability and mental wellbeing may reflect the fact that the measures of chronic disease used here only provide a partial picture of the extent of physical ill-health among the participants, but may also be due to the nature of these measures. Self ratings of health tend to be more strongly linked to feelings of happiness or satisfaction than more objective measures, such as disease checklists. [39], [40].

Examination of the unadjusted effect estimates from individual cohorts shows that associations between cognitive ability in later life and, more particularly, change in cognition since age 11 years and mental wellbeing tended to be stronger in the two national birth cohorts than in the two Scottish cohorts. The reason for this is unclear. It might be that the association between change in cognitive function since childhood and mental wellbeing gets weaker at older ages, although this seems unlikely to be the whole explanation as the associations in NCDS and NSHD were strikingly similar despite the fact that their members were born 13 years apart. It may be that the size of the Scottish samples meant we lacked the statistical power to detect what appears to be a modest association.

The main strength of our study is the availability of data from four cohorts, two of them national birth cohorts, allowing us to explore the consistency of associations between lifetime cognitive ability on mental wellbeing in older people. It also has some weaknesses. Differences between the cohorts in the data collected on chronic disease meant that our adjustments for this were not identical in each cohort and may not have adequately captured the extent of physical illness. There were also some differences between the cohorts in the measures used to assess cognitive function, though in the two largest cohorts the measures used were either identical or very similar. Personality of the members of the four cohorts was assessed using three different personality inventories, but there is evidence that scores on measures of neuroticism and extraversion made using different personality inventories correlate strongly. [41], [51] Whereas cognitive function in later life was measured several years before mental wellbeing in three of the cohorts, thereby reducing the possibility that mental wellbeing might influence cognitive test performance, members of NCDS took tests of cognition and mental wellbeing on the same occasion. For one of the cohorts, NSHD, we used data on personality assessed in adolescence rather than in later life. There is however considerable evidence for stability of personality over time, [52] and the size of the associations between these adolescent measures of neuroticism and extraversion and later life measures of cognition and mental wellbeing were similar to those observed in the other cohorts. Finally, although the *I^2^* statistics for most of the overall effect estimates were between 0% and 36% implying low or moderate heterogeneity, 95% confidence intervals around the *I^2^* statistics tended to be wide because of the small number of our samples, ranging from 0% to as high as 89%, so the true extent of heterogeneity is uncertain.

In summary, we used data from four UK birth cohorts to investigate whether cognitive ability at age 11 years or in later life or lifetime cognitive change were associated with mental wellbeing, in older people. We found that people with greater cognitive ability, and those whose cognitive function had improved relative to their cognitive performance in childhood, had slightly higher mental wellbeing scores, but the effects were small, and seemed to be primarily accounted for by the personality trait neuroticism. Poorer cognitive ability in youth may be a risk factor for mental disorders many years later, [1]–[6] but these findings demonstrate that, at least in older people, it is not a strong predictor of mental wellbeing.
